# Anti-TIM3 chimeric antigen receptor-natural killer cells from engineered induced pluripotent stem cells effectively target acute myeloid leukemia cells

**DOI:** 10.1186/s12935-023-03153-9

**Published:** 2023-11-27

**Authors:** Phatchanat Klaihmon, Sudjit Luanpitpong, Xing Kang, Surapol Issaragrisil

**Affiliations:** 1 Siriraj Center of Excellence for Stem Cell Research, Faculty of Medicine Siriraj Hospital, Mahidol University, Bangkok, Thailand; 2 Blood Products and Cellular Immunotherapy Research Group, Faculty of Medicine Siriraj Hospital, Mahidol University, Bangkok, Thailand; 3 Division of Hematology, Department of Medicine, Faculty of Medicine Siriraj Hospital, Mahidol University, 2 Siriraj Hospital, Bangkoknoi, Bangkok, 10700 Thailand; 4 BDMS Center of Excellence for Hematology, Wattanosoth Cancer Hospital, Bangkok, Thailand

**Keywords:** Induced pluripotent stem cells, Natural killer cells, Chimeric antigen receptor, CAR, TIM3, Acute myelogenous leukemia, Immunotherapy

## Abstract

**Background:**

Acute myeloid leukemia (AML) is a clonal malignant disorder which originates from a small number of leukemia-initiating cells or leukemic stem cells (LSCs)—the subpopulation that is also the root cause of relapsed/refractory AML. Chimeric antigen receptor (CAR)-T cell therapy has proved successful at combating certain hematologic malignancies, but has several hurdles that limit its widespread applications. CAR-natural killer (NK) cells do not carry the risk of inducing graft-versus-host disease (GvHD) frequently associated with allogeneic T cells, thereby overcoming time-consuming, autologous cell manufacturing, and have relatively safer clinical profiles than CAR-T cells. The present study aimed to generate anti-TIM3 CAR-NK cells targeting LSCs from a clonal master induced pluripotent stem cells engineered with the third-generation anti-TIM3 CAR.

**Methods:**

A clonal master umbilical cord blood NK-derived induced pluripotent stem cell (iPSC) line, MUSIi013-A, was used as a starting cells for engineering of an anti-TIM3 CAR harboring TIM3 scFv fragment (clone TSR-022), CD28, 4-1BB, and CD3ζ signaling (CAR-TIM3). The established CAR-TIM3 iPSCs were further differentiated under serum- and feeder-free conditions into functional CAR-TIM3 NK cells and tested for its anti-tumor activity against various TIM3-positive AML cells.

**Results:**

We successfully established a single-cell clone of CAR-TIM3 iPSCs, as validated by genomic DNA sequencing as well as antibody and antigen-specific detection. We performed thorough iPSC characterization to confirm its retained pluripotency and differentiation capacity. The established CAR-TIM3 iPSCs can be differentiated into CAR-TIM3 NK-like cells, which were further proven to have enhanced anti-tumor activity against TIM3-positive AML cells with minimal effect on TIM3-negative cells when compared with wild-type (WT) NK-like cells from parental iPSCs.

**Conclusions:**

iPSCs engineered with CARs, including the established single-cell clone CAR-TIM3 iPSCs herein, are potential alternative cell source for generating off-the-shelf CAR-NK cells as well as other CAR-immune cells. The feasibility of differentiation of functional CAR-TIM3 NK cells under serum- and feeder-free conditions support that Good Manufacturing Practice (GMP)-compliant protocols can be further established for future clinical applications.

**Supplementary Information:**

The online version contains supplementary material available at 10.1186/s12935-023-03153-9.

## Background

Acute myeloid leukemia (AML) is a clonal malignant disorder which originates from a small number of leukemia-initiating cells or leukemic stem cells (LSCs). LSCs have capacity of self-renewing and propagating leukemic progenitors (LPCs) that actively divide to produce a large number of immature leukemic blasts, resulting in an impaired normal hematopoiesis [1–3]. In patients with AML, the hematopoietic tissues comprise both residual normal hematopoietic stem cells (HSCs) and LSCs, which is supported by the recovery of normal hematopoietic cells after chemotherapy treatment [4]. It has been demonstrated that the AML-LSCs reside mainly in the CD34^+^CD38^−^ fraction, which are the mutual phenotypic marker of normal HSCs [2]. Regarding the therapeutic options for patients with AML, the allogeneic HSC transplantation following myeloablative conditioning serves as the only potentially curative approach [5, 6] but a few number of patients show remarkable outcome due to the treatment toxicity and persistence of residual LSCs [7, 8]. To target and eradicate the LSCs in AML patients without killing normal HSCs, there is a critical need of unique biomarker to distinguish between them. There are several reports attempting to use preferential markers such as CD33, CD44, CD47, CD96, CD123, CLL-1 and TIM3 to separate AML-LSCs from normal HSCs [9, 10]. Among them, TIM3 (encoded by *HAVCR2*) is an interesting candidate and has gained much attraction because it is expressed in both CD34^+^CD38^−^ LSCs and CD34^+^CD38^+^ LPCs in the vast majority of AML subtypes but not in normal HSC counterpart [11, 12].

Chimeric antigen receptor (CAR)-T cell therapy has proved successful at combating certain hematologic malignancies, particularly CD19-positive B-cell malignancies, including relapsed/refractory acute lymphoblastic leukemia (ALL), diffuse large B-cell lymphoma (DLBCL), and mantle cell lymphoma (MCL) [13, 14]. Incorporation of tumor antigen-specific CAR to T cells enhances their ability to recognize the target tumor cells in vivo, which then activate signaling through CAR to induce CAR-T cell proliferation, cytokine secretion, and target killing. However, major limitations of CAR-T cell therapy include life-threatening complications, including graft-versus-host disease (GvHD), which may be caused by allogeneic CAR-T cells, and other adverse effects, e.g., cytokine release syndrome (CRS) and neurological toxicity [15–18]. Additionally, time-consuming and high-cost of autologous CAR-T preparation is also an important concern. To avoid these problems, natural killer (NK) cells, which have shorter life-span and exert major histocompatibility complex (MHC)-unrestricted cytotoxicity, are the next candidate cells for armoring with CARs [19, 20]. There are several reports of using ex vivo expanded NK cells collected from primary sources, i.e., cord blood and peripheral blood, for cancer immunotherapy but their proliferation rate and cytotoxicity could be declined during prolonged culture, which may vary among donors [21, 22]. Derivation of NK cells from clonal master pluripotent stem cells, especially induced pluripotent stem cells (iPSCs), should be considered to generate standardized, off-the-shelf homogeneous NK population expressing NK cell-specific receptors with potent cytotoxicity against tumor cells [23, 24].

iPSCs are an ideal starting material for CAR-related immune cell therapy, due to its ease of genetic manipulation and limitless expansion during iPSC stage [25, 26]. Previously, we successfully differentiated NK-derived iPSCs (NK/iPSCs), engineered with or without a third-generation anti-CD19 CAR, reprogrammed from CD3^−^CD56^+^CD16^+^ umbilical cord blood (UCB)-derived NK cells into functional NK cells to target B cell malignancies [27]*.* In the present study, we designed and constructed a third-generation anti-TIM3 CAR using the similar framework as an anti-CD19 CAR, which consists of scFv fragment, CD3ζ signaling domain, and costimulatory domains CD28 and 4-1BB. Upon introduction of anti-TIM3 CAR into NK/iPSCs, we performed single-cell clone selection and further described its characterization and efficient differentiation into functional anti-TIM3 CAR-NK cells.

## Results

### Engineering NK/iPSCs with anti-TIM3 CAR

**Fig. 1:**
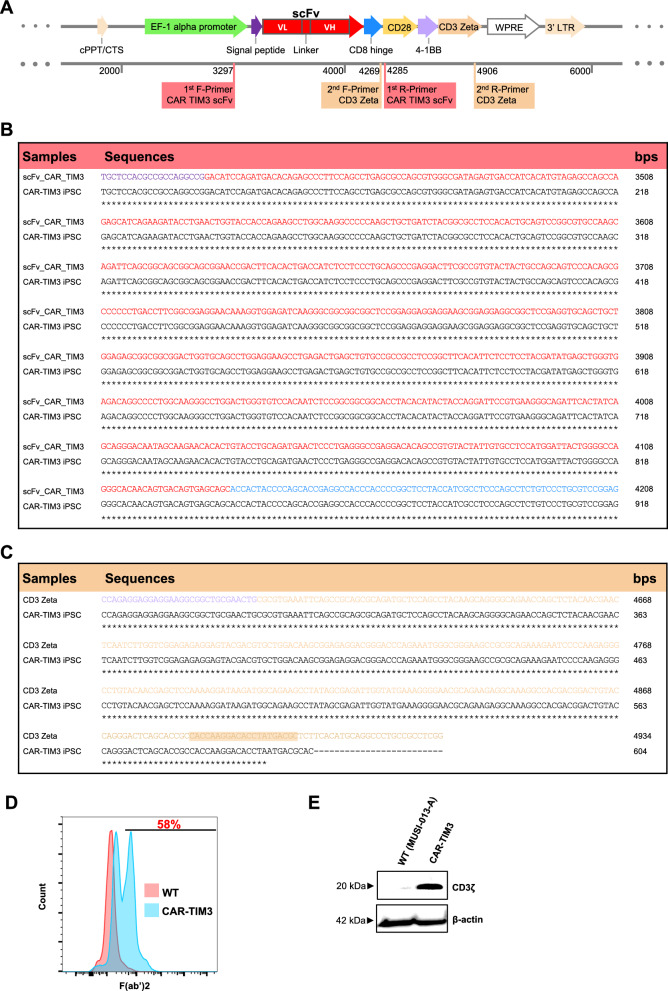
Construction of CAR-TIM3 and its introduction into iPSCs. **A **(**Upper**) Diagram of the third-generation CAR-TIM3 construct harboring signal peptide, anti-TIM3 scFv fragment, CD8 hinge, CD28, 4-1BB, and CD3ζ, under the control of EF-1α promoter. (**lower**) Two sets of primers used for DNA sequencing and its predicted target regions are shown. **B**, **C** DNA sequencing results of CAR-TIM3 iPSCs (clone 5E1) spanning over scFv fragment (**B**) and CD3ζ (**C**). **D** CAR expression on the surface of CAR-TIM3 iPSCs as evaluated by flow cytometry using anti-F(ab′)2 antibody. **E** Western blot analysis of CD3ζ, which is a CAR signaling molecule

We previously generated the single-cell clone NK/iPSCs, registered in Human Pluripotent Stem Cell Registry (hPSCreg^®^; accessible at http://hpscreg.eu) as MUSIi013-A, by using three non-integrating episomal reprogramming plasmids encoding KLF4, OCT3/4, SOX2, L-MYC, LIN28 and shRNA against *TP53* and an extra plasmid encoding EBNA1 to enhance the reprogramming efficiency [28]. Briefly, all plasmids were introduced into UCB-derived NK cells by 4D-nucleofection. Following a single-cell clone selection and iPSC characterization, the MUSIi013-A NK/iPSCs was established and further transduced with lentiviral particles carrying the third-generation anti-TIM3 CAR illustrated in Fig. 1a (upper). The structure of CAR construct is anti-TIM3 scFv fragment (clone TSR-022) comprising heavy and light chains joined to co-stimulatory molecules CD28, 4-1BB and CD3ζ chain via a CD8 hinge region with a signaling peptide sequence (MALPVTALLLPLALLLHAARP). After lentiviral transduction, single-cell clone selection was performed by plating the single cells of CAR-TIM3 iPSCs onto irradiated human fibroblasts in NutriStem medium supplemented with a small molecule cocktail of 4 inhibitors (SMC4) consisting of PD0325901 (MEK inhibitor), SB431542 (TGFβ inhibitor), Thiazovivin (ROCK inhibitor) and CHIR99021 (GSK3 inhibitor) in a 96-well plate. Emerging iPS colonies were cultured and expanded, after which clone 5E1 was picked for thorough characterization. Firstly, genomic DNA sequencing was performed to confirm the integration of anti-TIM3 CAR using two sets of primers targeting scFV and CD3ζ (Fig. 1a, lower). Identical nucleotide sequences between CAR construct and CAR-TIM3 iPSCs verified the successful insertion of CAR transgene (Fig. 1b, c). Secondly and thirdly, the detection of F(ab′)2 fragment by flow cytometric analysis (Fig. 1d) and chimeric CD3ζ by Western blotting (Fig. 1e) confirmed the CAR expression.

### Pluripotent characterization and multi-lineage differentiation of CAR-TIM3 iPSCs

**Fig. 2:**
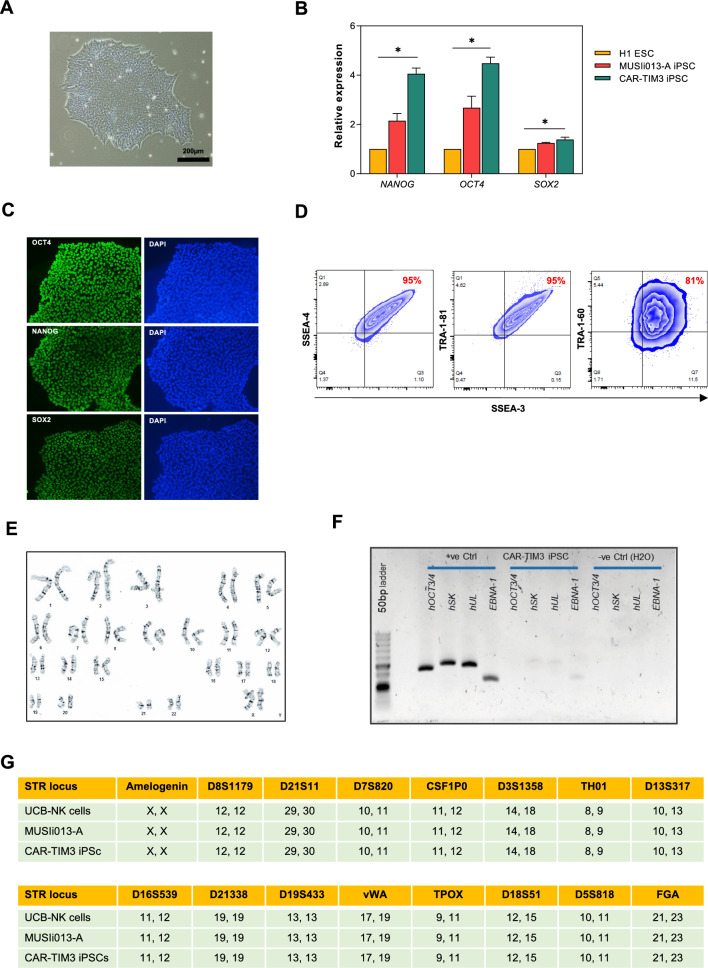
Characterization of CAR-TIM3 iPSCs. **A** Representative micrograph of CAR-TIM3 iPSC colony. **B** Expression of *NANOG*, *OCT4*, and *SOX2* transcripts as quantitative measurement by qPCR analysis. Data are mean ± SEM (n = 3). *p < 0.05 versus H1 ESC; unpaired *t*-test. **C** Representative micrographs of immunofluorescence staining showing pluripotency markers OCT4, NANOG, and SOX2 proteins. **D** Flow cytometric analysis of SSEA-3, SSEA-4, TRA-1-60 and TRA-1-81 positive cells. **E** Karyotypic analysis as determined by G-banding assay. **F** Evidence of the loss of episomal transgenes detected by PCR and gel electrophoresis. **G** STR analysis comparing a total of 16 loci between starting UCB-NK cells, MUSIi013-A NK/iPSCs and CAR-TIM3 iPSCs

Further, CAR-TIM3 iPSCs (clone 5E1) was fully characterized for its pluripotency by determining the expression of stem cell-related genes and proteins, including OCT4, NANOG and SOX2 by quantitative real-time PCR (qPCR) analysis and immunofluorescence staining (Fig. 2b, c). In addition, the pluripotent surface markers such as SSEA-3, SSEA-4, TRA-1-60 and TRA-1-81 were confirmed by flow cytometric analysis (Fig. 2d). Figure 2e shows that CAR-TIM3 transgene did not cause chromosomal abnormality as determined by karyotyping at passage 25. The absence of reprogramming transgenes was confirmed once again by PCR (Fig. 2f). Short tandem repeat (STR) analysis confirmed that CAR-TIM3 iPSCs share a 100% DNA profile match with their parental MUSIi013-A iPSCs and the starting UCB-derived NK cells (Fig. 2g).

**Fig. 3:**
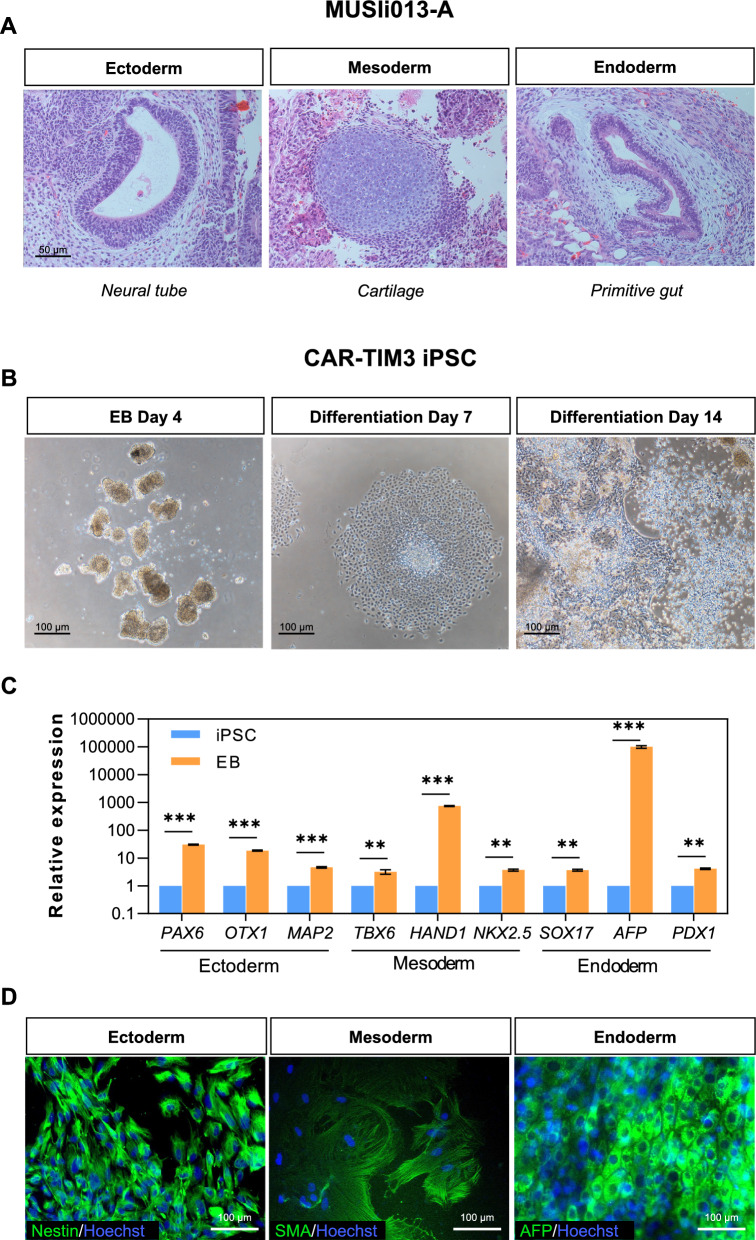
Differentiation potential of MUSIi013-A NK/iPSCs and its subclone CAR-TIM3 iPSCs. **A** Differentiation potential of parental MUSIi013-A NK/iPSCs was first performed by teratoma formation in SCID mice. Representative micrographs of H&E stained sections of ectoderm, mesoderm, and endoderm. **B–D** Differentiation potential of CAR-TIM3 iPSCs as evaluated by EB formation. Morphology of floating EBs on days 4, 7 and 14 of differentiation. **C** mRNA expression of tri-lineage differentiation markers: *PAX6, OTX1*, and *MAP2* for ectoderm; *TBX6, HAND1*, and *NKX2.5* for mesoderm; and *LEFTY1* and *AFP* for endoderm. Data are mean ± SEM (n = 3). **p < 0.01, ***p < 0.001 versus non-differentiated CAR-TIM3 iPSCs; unpaired *t*-test. **D** Representative micrographs of immunofluorescence staining of tri-lineage differentiation markers

Apart from its pluripotency characterization, the differentiation potential of CAR-TIM3 iPSCs into three embryonic germ layers must be performed. As the parental MUSIi013-A iPSCs were proven to form teratoma with ectodermal, mesodermal, and endodermal morphology in vivo (Fig. 3a), the in vitro spontaneous embryoid body (EB) formation assay was chosen to confirm once again the differentiation potential of the subclone CAR-TIM3 iPSCs. Figure 3b compares morphology of the floating EBs derived from CAR-TIM3 iPSCs on day 4 with morphology of the same EBs, which spontaneously attached to the culture plate, on days 7 and 14 of differentiation. The significant upregulation of representative genes for ectodermal (*PAX6, OTX1* and *MAP2*), mesodermal (*TBX6, HAND1* and *NKX2.5*) and endodermal (*LEFTY1* and *AFP*) lineages in EBs on days 14 of differentiation were observed when compared with the non-differentiated CAR-TIM3 iPSCs (Fig. 3c). Immunofluorescence staining further confirmed the expression of markers for endoderm (α-fetoprotein, AFP), mesoderm (smooth muscle actin, SMA), and ectoderm (Nestin), verifying the three-germ layer differentiation (Fig. 3d). After completing thorough iPSC characterization, the established CAR-TIM3 iPSC line was registered in hPSCreg^®^ as MUSIi013-A-2.

### Hematopoietic cell induction from CAR-TIM3 iPSCs

**Fig. 4:**
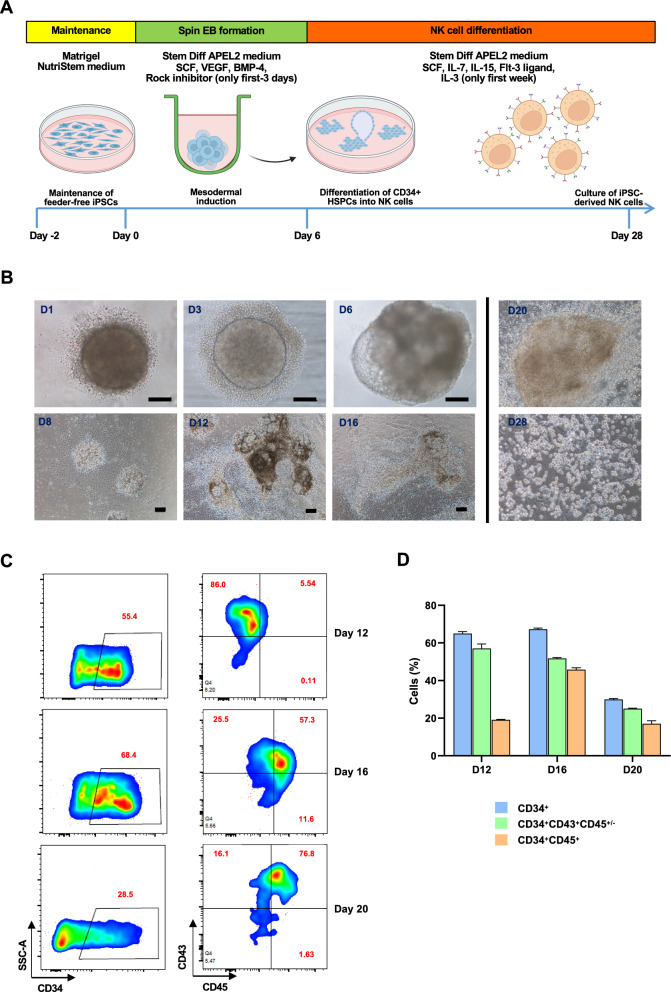
Generation of spin EBs and induction of HSPCs from CAR-TIM3 iPSCs. **A** Schematic diagram illustrating a two-stage protocol for the differentiation of NK cells from iPSCs under serum- and feeder-free conditions. **B** Morphology of generated EBs on various days of culture. iPS sacs were observed on day 12 onwards and completely disappeared on day 28. **C**, **D** Representative flow cytometric analysis of HSPC markers CD34, CD43, and CD45 in floating cells on days 12, 16, and 20 of culture

We aimed to differentiate functional NK cells expressing anti-TIM3 CAR, shorten as CAR-TIM3 NK cells, from CAR-TIM3 iPSCs using xeno-free conditions, i.e., serum-free media and feeder-free culture system. First, spin EB formation was used to generate mesoderm progenitors. Then, the derived hematopoietic stem/progenitor cells (HSPCs) were further differentiated into NK cells, as schematically outlined in Fig. 4a. By modifying the differentiation protocol published by Lupo et al. [29], EBs were formed from 5000 cells of iPSCs after dispersal into single cells by Accutase enzyme. At day 6, EBs were transferred onto Matrigel-coated plate and culture medium were changed from hematopoietic differentiation medium to NK cell differentiation medium. Morphology of spin EBs and attached EBs at various days were shown in Fig. 4b to demonstrate their dynamic changes. Floating cells at days 12, 16 and 20 were harvested and subjected to flow cytometric analysis of HSPC surface markers, including CD34, CD43 and CD45. At the early phase of differentiation, the floating cells were defined to be CD34^+^CD43^+^ HSPCs with CD45^+/−^. Notably, the CD34^−^ cells were found to be either CD43^+^ or CD45^+^ (Additional file 1: Fig. S1), indicating that all the floating cells were hematopoietic cells. At the later stage (day 20), they were shifted to be the more mature CD34^+^CD43^+^CD45^+^ cells, and the number of floating cells were gradually decreased. Repopulation of floating cells were observed at the 4th week of differentiation.

### NK cell differentiation from the iPSC-derived HSPCs

**Fig. 5:**
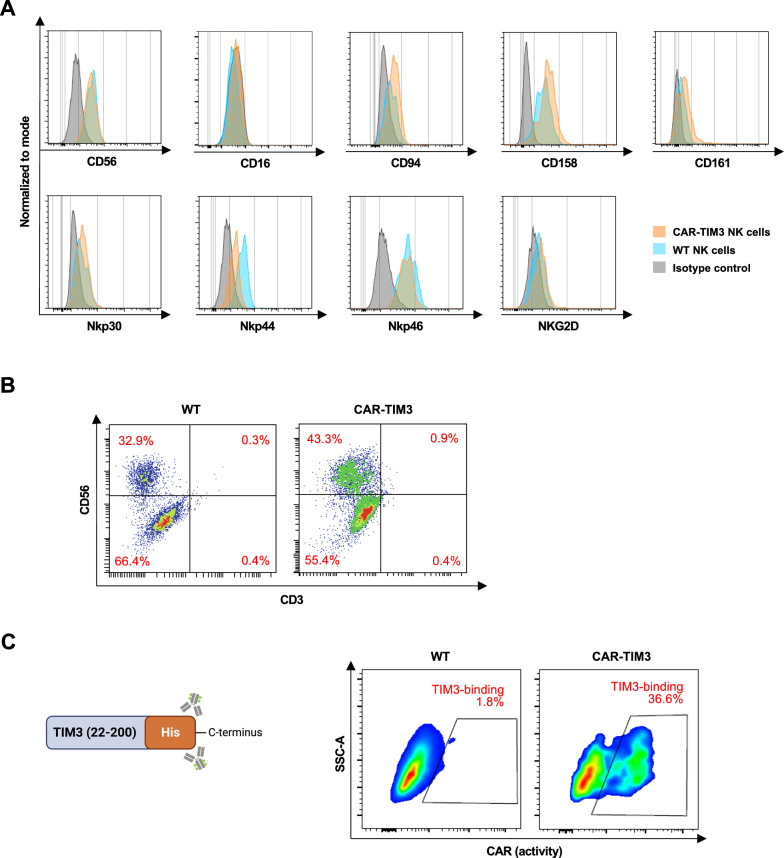
Immunophenotypic profiles of NK-like cells derived from WT and CAR-TIM3 iPSCs. **A** Flow cytometric analysis of typical NK surface markers CD16, CD56, and NK cell-related receptors, including CD94, CD158, CD161, Nkp30, Nkp44, Nkp46 and NKG2D on NK-like cells derived from parental WT- and CAR-TIM3 iPSCs on day 30 of culture. **B** Flow cytometric analysis of CD3 and CD56 confirms the absence CD3^+^ CD56^−^ T-like and CD3^+^ CD56^+^ NKT-like cells. **C** Anti-TIM3 CAR expression in the differentiated CAR-TIM3 NK-like cells as evaluated by flow cytometry based on its binding activity to specific antigen His tag-rhTIM3 (23–200)

At day 28 onwards, floating cells from iPSC-derived HSPCs, either MUSIi013-A (hereafter called WT iPSCs) or CAR-TIM3 iPSCs, were collected and subjected to flow cytometric analysis of NK cell-specific markers/receptors, including CD56, CD94, CD158, CD161, Nkp30, Nkp44, Nkp46, NKG2D, and CD16. Our modified differentiation protocol was capable of producing either WT or CAR-TIM3 NK-like cells as defined by the expression of most of the NK cell marker/receptors, except for CD16 (Fig. 5a). Figure 5b shows that the majority (> 98%) of differentiated cells were CD3^−^ cells, thereby confirming the absence of WT or CAR-TIM3 T- and NKT-like cells under this NK differentiation protocol. Next, we confirmed that the differentiated CAR-TIM3 NK-like cells, but not WT NK-like cells had the ability to bind to recombinant human TIM3 protein (Fig. 5c), indicating that CAR-TIM3 NK-like cells retained anti-TIM3 CAR expression, which showed high specificity.

### Anti-leukemia activity of CAR-TIM3 NK cells

**Fig. 6:**
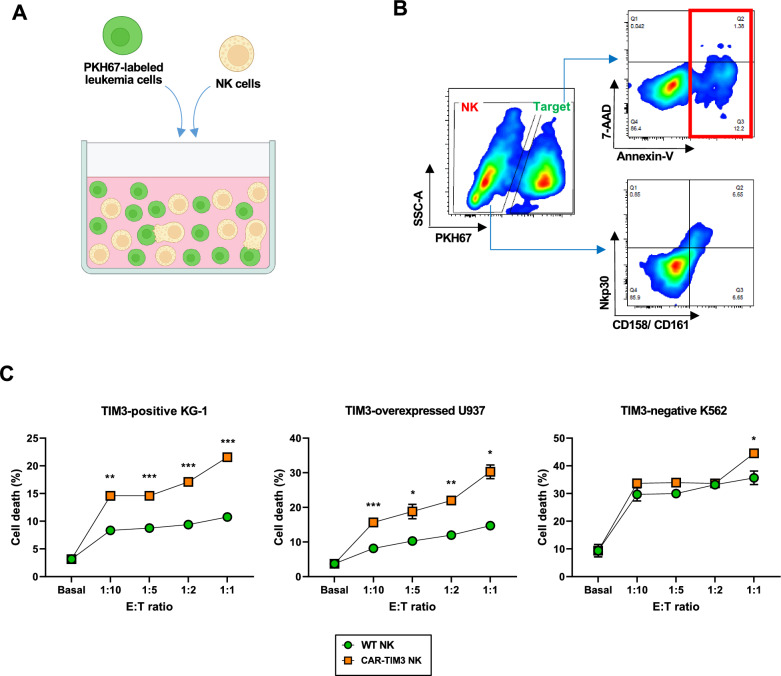
CAR-TIM3 NK-like cells selectively induced cell death of TIM3-positive target leukemia cells. **A** Schematic diagram of NK cytotoxicity assay showing the labeling of target tumor cells with PKH67 dye before exposure to effector cells at various E:T ratios. **B** Flow cytometric gating strategy to specifically detect cell death in PKH67-labeled target tumor cells and NK cell markers in effector NK-like cells. **C** Percentages of total tumor cell death, comprising Annexin V- and/or 7-AAD-positive cells, in TIM3-negative K562 cells and TIM3-positive KG-1 cells and TIM3-overexpressed U937 cells at indicated E:T ratio were plotted. Data are mean ± SEM (n = 3). *p < 0.05; **p < 0.01; and ***p < 0.001 versus WT NK-like cells in the same condition; unpaired *t*-test

As mentioned earlier, TIM3 is generally expressed in the CD34^+^ LSCs and LPCs in AML. Herein, we tested the cytotoxicity of the differentiated CAR-TIM3 NK-like cells against TIM3-positive AML cells, including CD34^+^ KG-1 cells and CD34^−^ U937 cells, which were introduced with TIM3 transgene, hereafter called TIM3-overexpressing U937 cells (Additional file 1: Fig. S2). Chronic myelogenous leukemia (CML)-derived TIM3-negative K562 cells were used as a negative control. In this assay, target tumor cells were pre-labeled with PKH67 green fluorescence dye and co-cultured with either WT- or CAR-TIM3 NK-like cells at the different effector:target (E:T) ratios of 1:10, 1:5, 1:2 and 1:1 for 4 h, as schematically illustrated in Fig. 6a. PKH67-labeled target tumor cells were then evaluated for cell death in using Annexin V/7-AAD assay, while the non-PKH67 labeled NK-like cells were determined for the expression of certain NK cell markers, including CD158, CD161 and Nkp30 (Fig. 6b). Figure 6c shows that CAR-TIM3 NK-like cells had superior anti-leukemia activity against TIM3 positive KG-1 and TIM3-overexpressing U937 cells when compared with WT NK-like cells, while exerting a minimal activity on K562 cells. These results indicate that our CAR-TIM3 NK-like cells specifically targeted and killed TIM3-positve AML cells.

### Expression of NK cell receptors upon tumor exposure

**Fig. 7:**
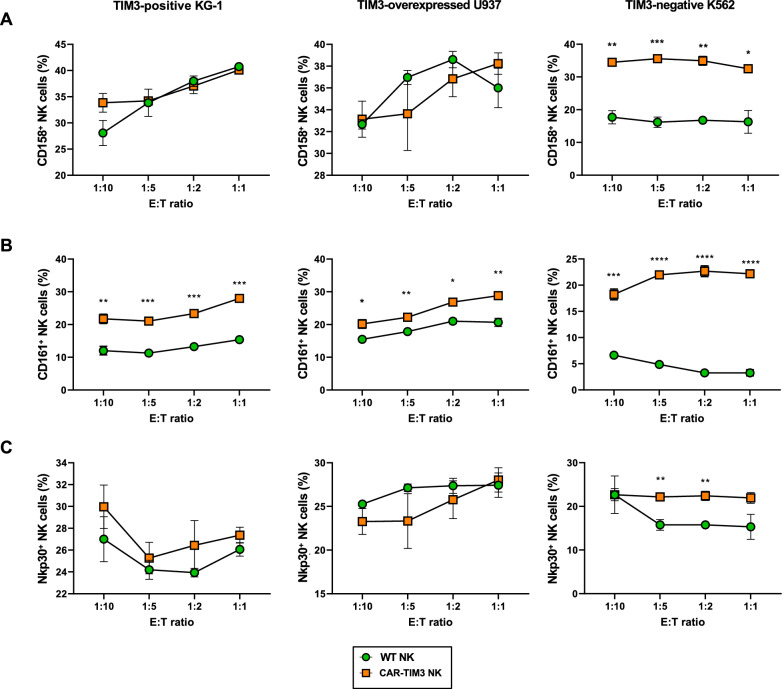
Expression of NK cell-related receptors in WT and CAR-TIM3 NK-like cells upon co-incubation with leukemia cells. **A–C** Surface CD158 (**A**), CD161 (**B**), and Nkp30 (**C**) in WT and CAR-TIM3 NK-like cells in response to K562, KG-1, and TIM3-overexpressed U937 cells at various E:T ratios were evaluated by flow cytometric analysis, and their percentages were plotted. Data are mean ± SEM (n = 3). *p < 0.05; **p < 0.01; ***p < 0.001 and ****p < 0.0001 versus WT NK-like cells under the same conditions; unpaired *t*-test

To confirm that the anti-leukemia activity of our differentiated NK-like cells was mediated via the activation of NK cell receptors, we evaluated the expression of CD158, CD161 and Nkp30 in NK-like cells upon tumor exposure, as depicted in Fig. 6b. Figure 7a–c reveals that exposure of WT and CAR-TIM3 NK-like cells to different target tumor cells resulted in different NK activation profiles. While CAR-TIM3 NK-like cells significantly upregulated an activating receptor CD161 in response to all tested TIM3-positive and negative leukemia cells when compared with WT NK-like cells, its effects on Nkp30 and CD158 were observed only in response to TIM3-negative K562 cells. CD158 is an NK inhibitory receptor, thus it might play a role in inhibiting NK cytotoxicity in K562 cells. Although it is difficult to address how CAR-TIM3 NK-like cells strengthen NK activating signals and selectively killed TIM3-positive cells, these data support that the differentiated NK-like cells, from both WT and CAR-TIM3 iPSCs, were functional NK cells.

## Discussion

CAR-NK cells are a potential competitor of CAR-T cells, as CAR-NK cells could overcome the major limitations of CAR-T cells that restrict its wider clinical applications [30, 31]. Thus far, various types of CAR-T and CAR-NK cells have been tested for the treatment of patients with hematological malignancies, particularly B-cell lymphoma and ALL. However, relatively less studies focus on the use of CAR-T or CAR-NK cells for targeting AML, due to the heterogeneity of AML cells that make them a moving target. A major challenge in AML therapy is the majority of AML patients experience a relapse even after complete remission, which is linked to an increased number of LSCs. The quiescent LSCs are known to be resistant to conventional chemotherapy and radiation [32]. Herein, we first introduced the third-generation CAR to target TIM3, which is highly expressed in primitive LSCs and LPCs, into human iPSCs to subsequently produce CAR-TIM3 NK-like cells in order to eradicate the root cause of relapsed/refractory AML.

Various allogeneic NK cell sources with different pros and cons can be used to generate off-the-shelf CAR-NK cell products. NK cells from either peripheral blood or cord blood display potent tumor-killing ability across several cancer cell types, but their proportion in circulating lymphocytes are relatively small and hence they are required to activated and expanded [33, 34]. NK-92 cell line is another important cell source of ongoing clinical trial [35]. Earlier, we have proposed the idea of generating a library of CAR-NK-92 cells targeting various tumor antigens [36]. However, the major limitation of CAR-NK-92 cells is the need of irradiation prior to clinical use for safety reasons, making them having a short life cycle in vivo.

Given that iPSCs have a capacity in differentiating into most cell types in the body, excluding germ cells, and a limitless life-span with an ease of genetic manipulation [25, 26], they would be the great starting cell source to generate functional NK cells with proven anti-tumor activity [37, 38]. By utilizing these advantages of iPSCs, we created a single-cell clone of CAR-TIM3 iPSCs from a clonal master MUSIi013-A line, which was reprogrammed from UCB-NK cells, with the anticipation that they could be efficiently differentiated into CAR-TIM3 NK cells from the retained epigenetic memory. In the present study, we demonstrated the ability of the CAR-TIM3 NK/iPSCs to differentiate into functional NK-like cells, which were positive for multiple key NK markers/receptors, except for CD16. The differentiated CAR-TIM3 NK-like cells were highly cytotoxic toward TIM3-positive leukemia cells through their endogenous NK signaling mediated by the balance of NK activating and inhibitory receptors and through the CAR-directed cytotoxicity. We postulated that the engineered CAR-TIM3 activated NK signaling via p38 and ERK, but not NF-κB, JNK and Akt, intracellular signaling pathways via protein phosphorylation (Additional file 1: Fig. S3). These differentiated cells, however, lack of antibody-dependent cellular cytotoxicity (ADCC) due to the absence of CD16 or FcγIII receptor, which is in agreement with other findings [39, 40]. To solve the issue of low CD16 in iPSC-derived NK cells, the introduction of CD16 transgene to enhance its anti-tumor activity has been considered. For example, transgenes comprising a non-cleavable, high-affinity CD16 and an interleukin-15 fusion protein has been used to augment the survival and function of FT596, the first ever iPSC-derived NK cells targeting CD19-positive lymphoid leukemias underwent a clinical trial (NCT04245722) [41]. Regarding killing mechanisms, CAR-NK cells have advantage over CAR-T cells as they could still target tumor cells even in the emergence of antigen escape in tumor cells from the loss of target antigen.

In addition to being the target tumor antigen that specifically expressed in AML-LSCs and LPCs, TIM3 is also, interestingly, an exhaustion marker of various immune cells, including T and NK cells [42–44]. Earlier, sabatolimab, a monoclonal antibody targeting TIM3, has been developed and underwent phase Ib clinical trial (NCT03066648) in combination with hypomethylating agents, i.e., decitabine and azacitidine, in patients with AML and high-risk myelodysplastic syndromes (MDS) [45]. It is postulated that blockade of TIM3 by sabatolimab may restore immune function while also directly targeting LSCs and blasts. Likewise, targeting exhausted immune cells by CAR-TIM3 NK-like cells may be beneficial to cancer treatment, which warrants further investigations.

## Conclusion

Taken together, our findings provide promising evidence on the generation of functional CAR-TIM3 NK-like cells for targeting primitive AML cells from a single-cell clonal master NK/iPSCs under serum-free and xeno-free conditions, which could be further developed into the Good Manufacturing Practice (GMP)-compliant protocols. Further studies should focus on improving differentiation efficiency at the stages of mesoderm/hematopoietic cell induction and/or NK commitment and on preclinical evaluation of differentiated CAR-TIM3 NK-like cells in animals. Additionally, strategies to enable long-term expansion of the differentiated CAR-TIM3 NK-like cells is important for further clinical applications. The residual undifferentiated iPSC contamination, which may be eliminated by irradiation, should be monitored in clinical iPSC-derived products as they may pose an intrinsic risk of tumorigenicity in patients.

## Materials and methods

### Cell culture

Human iPSCs were maintained on Matrigel-coated plates (Corning, Corning, New York, USA) in NutriStem XF medium (Sartorius, Göttingen, Germany) and sub-passaged every 2–3 days using Versene solution (Gibco, Waltham, MA, USA). Human leukemia-derived cells used in this study, including KG-1, U937 and K562 cells were obtained from American Type Culture Collection (ATCC; Manassas, VA, USA) and cultivated in RPMI1640 medium supplemented with 10% fetal bovine serum (FBS), L-glutamine and antibiotics (Gibco) in CO_2_ humidified atmosphere at 37 °C. Cells that were positive for mycoplasma contamination, as evaluated by MycoAlert PLUS Mycoplasma Detection Kit (Lonza, Basel, Switzerland), were discarded.

### Viral production and transduction

For CAR-TIM3 transduction, the third-generation, lentiviral CAR construct (Creative Biolabs, Shirley, NY, USA), schematically depicted in Fig. 1a (upper), were inoculated in HEK293FT cells (Thermo Fisher Scientific, Waltham, MA, USA) in conjunction with VSV-G and dR8.2 plasmids (Addgene, #8454 and #8455) at a ratio of 10:1:4 using Lipofectamine 3000 (Thermo Fisher Scientific). Parental, single-cell clone iPS line, MUSIi-013-A, were dissociated into a single cell suspension using Accutase (Stemcell Technologies, Vancouver, BC, Canada) before seeding onto Matrigel-coated plate for 24 h, and transduced with concentrated lentiviral particles. Then, a single-cell clone dilution of transduced iPSCs was performed in a 96-well plate pre-seeding with irradiated human foreskin fibroblasts in the presence of SMC4 consisting PD0325901 (Sigma Aldrich, St. Louis, MO, USA), CHIR99021, Thiazovivin, and SB431542 (Stemcell Technologies). Emerging colonies were picked up for further iPSC characterization.

TIM3 overexpression in U937 cells was performed using pQCXIX-Myc-hTIM3 retroviral plasmid (Addgene, #110893), in which retroviral particles were produced in Plat-A packaging cells (Cell Biolabs, San Diego, CA). At 3-day post-transduction, U937 cells were expanded and sorted for TIM3-positive cells using FACS Aria Fusion cell sorter (BD Biosciences, San Jose, CA, USA).

### Genomic DNA sequencing

Genomic DNA was isolated using a PureLink Genomic DNA Mini Kit (Invitrogen, Waltham, MA, USA). The target regions for DNA sequencing were amplified by PCR using Q5 High-Fidelity DNA Polymerase (New England Biolabs, Ipswich, MA, USA) with specific primers (Fig. 1a, lower), and the resulting PCR products were purified by a GenepHlow Gel/PCR kit (Geneaid, New Taipei City, Taiwan). A total of 0.2 μg PCR product was then used for DNA sequencing using ABI PRISM BigDye™ Terminator Cycle Sequencing Kit v3.1 (1st BASE, Singapore).

### Evaluation of CAR-TIM3 expression

CAR-TIM3 expression in iPSCs was determined by flow cytometry based on Fab fragments. Cells were incubated with FITC-conjugated anti-mouse-IgG, F(ab′)2 fragment antibody (F(ab′)2-FITC; Jackson ImmunoResearch, West Grove, PA, USA) for 30 min at 4 °C and analyzed using a BD FACS Canto (BD Biosciences). Western blotting of CD3ζ was used to confirm the successful introduction of CAR-TIM3.

CAR-TIM3 expression in the differentiated cells was evaluated by target antigen-based detection to ascertain its binding activity. Briefly, cells were incubated with 10 μg/mL rhTIM3 (23–200) protein with His tag to the C-terminus (Abcam, Cambridge, UK) for 1 h at 4 °C, followed by an incubation with FITC-conjugated anti-His tag antibody (His tag-FITC; Abcam) for 15 min at room temperature and flow cytometric analysis. Cells that were incubated with His tag-FITC, but not with rhTIM3, were used as a basal control. The percentage of cells that expressed CAR-TIM3 could be calculated from the subtraction of FITC-positive cells in the basal control from those with the target protein.

### Immunofluorescence staining

Cells were fixed in 4% paraformaldehyde for 20 min, permeabilized with 0.1% Triton X-100/PBS for 10 min, and blocked with 3% bovine serum albumin (BSA)/PBS for 1 h. The cells were incubated with primary antibodies in 1% BSA/PBS overnight at 4 °C, followed by secondary antibodies at room temperature for 1 h. Nuclei were counterstained with Hoechst 33,342 (Thermo Fisher Scientific) for 30 min at room temperature and visualized under fluorescence microscope (Eclipse Ti-U, Nikon, Tokyo, Japan) with NIS-Elements D Software (version 4.30.00; Nikon).

### Flow cytometry

For analysis of pluripotency markers, iPSCs were dissociated into single cells using TrypLE™ Select (Gibco), blocked with 10% human AB serum, stained with FITC-SSEA-3, PE-SSEA-4, Alexa Fluor 647-TRA-1-60, and PE-TRA-1-81 (BioLegend, San Diego, CA, USA), and analyzed by BD FACS Canto flow cytometer (BD Biosciences) with FlowJo software (V10.4.1).

For phenotypic analysis of HSPC induction and NK commitment, the cells were resuspended in FACS buffer (PBS with 2% BSA), incubated with antibody cocktail for 30 min at 4 °C in the dark, and analyzed by BD FACS Canto. The antibodies used in this study included PE-CD56, PerCP-CD45, APC-CD34 (BD Biosciences), FITC-CD16, PE/Cy7-CD43, FITC-Nkp46, PE-Nkp44, PerCP-CD94, APC-TIM3, PE/Cy7-KIR, Alexa Fluor 647-CD161 and BV605-Nkp30 (BioLegend).

### qPCR analysis

Total RNA was isolated using TRI Reagent^®^ (Molecular Research Center, Cincinnati, Ohio, USA) and converted to complementary DNA using the RevertAid First Strand cDNA synthesis kit (Thermo Fisher Scientific). The qPCR reactions were performed on the CFX384 Touch Real-Time PCR detection system (Bio-Rad, Hercules, CA, USA) using SYBR™ Select Master Mix (Thermo Fisher Scientific). The cycle parameters started with an activation step at 95 °C for 2 min, followed by 40 cycles of denaturation at 95 °C for 15 s and annealing/extension at 60 °C for 1 min.

### Karyotyping

The standard G-banded karyotyping was performed at the Department of Obstetrics and Gynecology, Faculty of Medicine Siriraj Hospital, Mahidol University. A total of 25 metaphases at a band resolution of 400–450 were analyzed.

### STR analysis

STR analysis was performed at the Department of Forensic Medicine, Faculty of Medicine Siriraj Hospital, Mahidol University. A total of 16 loci were tested.

### Teratoma formation

The iPSCs were treated with 10 μM of Y-27632 for 1 h prior to harvesting. Cells were resuspended at 5 × 10^6^ cells/100 μL of cold 30% (v/v) Matrigel^®^ Matrix (Corning) in NutriStem medium, and then implanted intramuscularly into 4-week-old female SCID mice (Nomura Siam International Co., Ltd, Thailand). At 4 weeks after transplantation, the mice were sacrificed and the teratomas were removed and fixed in 10% formalin overnight before being embedded in paraffin wax. Samples were sectioned and examined by hematoxylin and eosin (H&E) staining. All animal experiments were approved by the Siriraj Animal Care and Use Committee (Si-ACUC) of the Faculty of Medicine Siriraj Hospital, Mahidol University, Bangkok, Thailand (COA no. Si-ACUP 009/2561).

### Spontaneous in vitro differentiation via EB formation

The iPSCs were harvested into small clumps using 1 mg/mL Dispase (Gibco) and cultured on low attachment dishes in knockout DMEM supplemented with 20% knockout serum replacement, 2 mM GlutaMAX™, 0.1 mM MEM non-essential amino acid, 0.1 mM β-mercaptoethanol, 1 × insulin-transferrin-selenium-ethanolamine, and 100 U/mL penicillin/streptomycin (Gibco). The medium was replaced every other day. On day 7, EBs were transferred onto a 0.1% gelatin-coated plate and cultured at 37 °C and 5% CO_2_ for another 3 weeks.

### NK cell differentiation via spin EB formation

iPSCs were pre-treated with Rho kinase inhibitor (ROCKi, Stem Cell Technologies, Canada) for 1 h before single cell dissociation by Accutase. We have slightly modified the differentiation protocol from previous work by Lupo et al. [29]. Briefly, 5000 single cells were used to form EBs in a low-attachment round-bottom 96-well plate in hematopoietic differentiation medium comprising of 100 μL STEM diff APEL2 medium (Stem Cell Technologies) supplemented with 40 ng/mL SCF, 20 ng/mL each of VEGF and BMP4, and 10 μM ROCKi (for first 3 days). Plates were spin at 250 × g for 5 min to facilitate EB formation and placed in humidified normoxic CO_2_ incubator. Fresh medium was changed every 3 days by removal of 50 μL old medium and replacement with 100 μL fresh one. After 6 days, EBs were gently transferred onto Matrigel-coated plate in NK cell differentiation medium comprising of STEM Diff APEL2 medium, 20 ng/mL SCF, 20 ng/mL IL-7, 10 ng/mL IL-15, 10 ng/mL Flt-3 ligand, and 5 ng/mL IL-3 (only for first week). All cytokines used were obtained from R&D Systems (Minneapolis, MN, USA).

### Cytotoxicity assay of CAR-NK cells against leukemia cells

Leukemia cells as the target cells were pre-stained with fluorescent PKH67 dye (Sigma Aldrich) according to manufacturer’s instructions. NK cells were harvested and pre-stimulated with 500 U/mL IL-2 and 25 ng/mL IL-15 overnight before use. Co-incubation of NK cells and target cells were performed at indicated E:T ratios for 4 h at 37 °C in a total cells of 2 × 10^4^ cells/100 μL in a round-bottom 96-well plate. To determine the tumor killing activity of NK cells, cell mixtures were stained with PE-conjugated annexin-V and 7-AAD (BD Biosciences) in a binding buffer for 15 min at room temperature, and target cell death was evaluated only in PKH67-positive cells using BD FACS Canto (BD Biosciences).

### Statistical analysis

Data were represented as mean ± SEM and statistical analysis were performed using GraphPad Prism software (San Jose, CA, USA). The comparison between WT and CAR-TIM3 groups were performed using unpaired *t*-tests. Differences with *p*-values less than 0.05 were considered statistically significant: *p < 0.05, **p < 0.01, ***p < 0.001, and ****p < 0.0001.

### Supplementary Information

**Additional file 1: Fig. S1.** Representative flow cytometric analysis of HSPC markers CD34, CD43, and CD45 in floating cells on day 16 of NK cell differentiation culture. **Fig. S2.** Analysis of TIM3 surface expression by flow cytometric analysis. **Fig. S3.** Western blot analysis of various intracellular signaling pathways in CARTIM3 NK-92 cells in response to recombinant human TIM3 (200 μg/mL) at 4 hours in comparison to WT NK-92 cells.

## Data Availability

The datasets used and/or analyzed during the current study are available from the corresponding author on reasonable request.
