# Supplementary material for: Anti-TIM3 chimeric antigen receptor-natural killer cells from engineered induced pluripotent stem cells effectively target acute myeloid leukemia cells
Source: Cancer Cell Int. 2023 Nov 27;23:297. doi: 10.1186/s12935-023-03153-9 (PMC10680184; doi:10.1186/s12935-023-03153-9)
Supplement: Supplementary file 1 — Additional file 1: Fig. S1. Representative flow cytometric analysis of HSPC markers CD34, CD43, and CD45 in floating cells on day 16 of NK cell differentiation culture. Fig. S2. Analysis of TIM3 surface expression by flow cytometric analysis. Fig. S3. Western blot analysis of various intracellular signaling pathways in CARTIM3 NK-92 cells in response to recombinant human TIM3 (200 μg/mL) at 4 hours in comparison to WT NK-92 cells. [file 12935_2023_3153_MOESM1_ESM.pdf]

Anti-TIM3 chimeric antigen receptor-natural killer cells from engineered induced pluripotent stem cells effectively target acute myeloid leukemia cells

Phatchanat Klaihmon[1], Sudjit Luanpitpong[1,2], Xing Kang[1], Surapol Issaragrisil[1,3,4,*]

[1]Siriraj Center of Excellence for Stem Cell Research, Faculty of Medicine Siriraj Hospital, Mahidol University, Bangkok, Thailand

[2]Blood Products and Cellular Immunotherapy Research Group, Faculty of Medicine Siriraj Hospital, Mahidol University, Bangkok, Thailand

[3]Division of Hematology, Department of Medicine, Faculty of Medicine Siriraj Hospital, Mahidol University, Bangkok, Thailand

[4]BDMS Center of Excellence for Hematology, Wattanosoth Cancer Hospital, Bangkok, Thailand

Correspondence: Surapol Issaragrisil, Division of Hematology, Department of Medicine, Faculty of Medicine Siriraj Hospital, Mahidol University, 2 Siriraj Hospital, Bangkoknoi, Bangkok 10700, Thailand. Email: surapolsi@gmail.com

Supplementary information: Supplementary information accompanying this manuscript include Suppl Figs S1–S3.

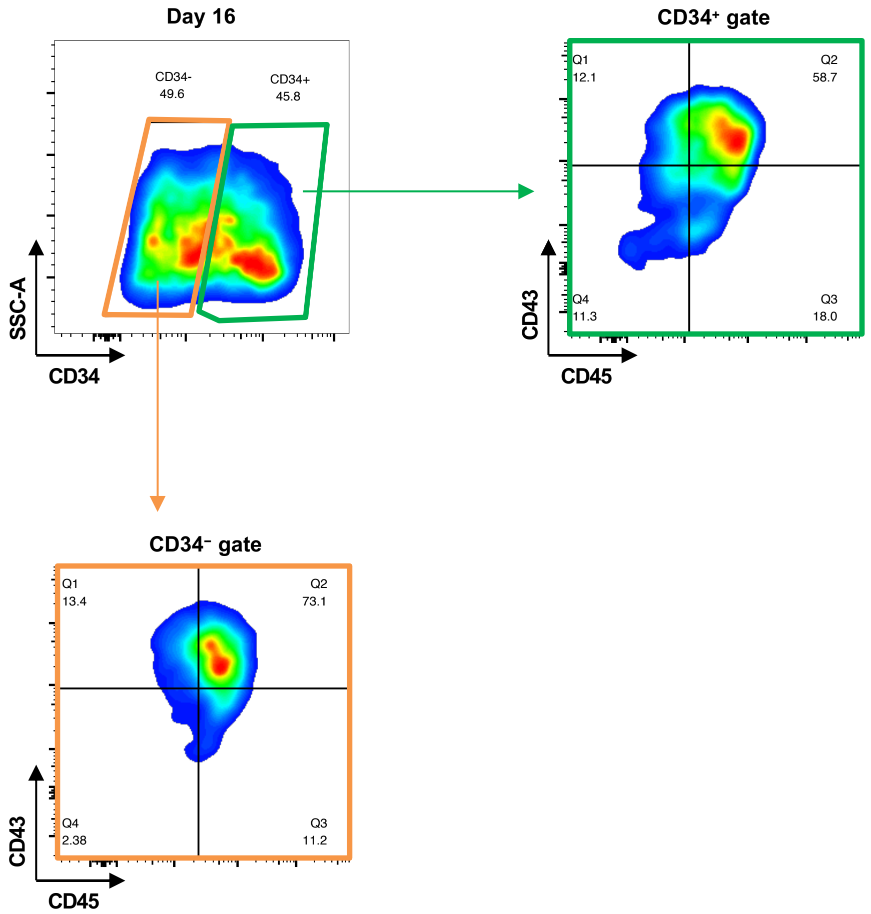

Suppl Fig S1 Representative flow cytometric analysis of HSPC markers CD34, CD43, and CD45 in floating cells on day 16 of NK cell differentiation culture.

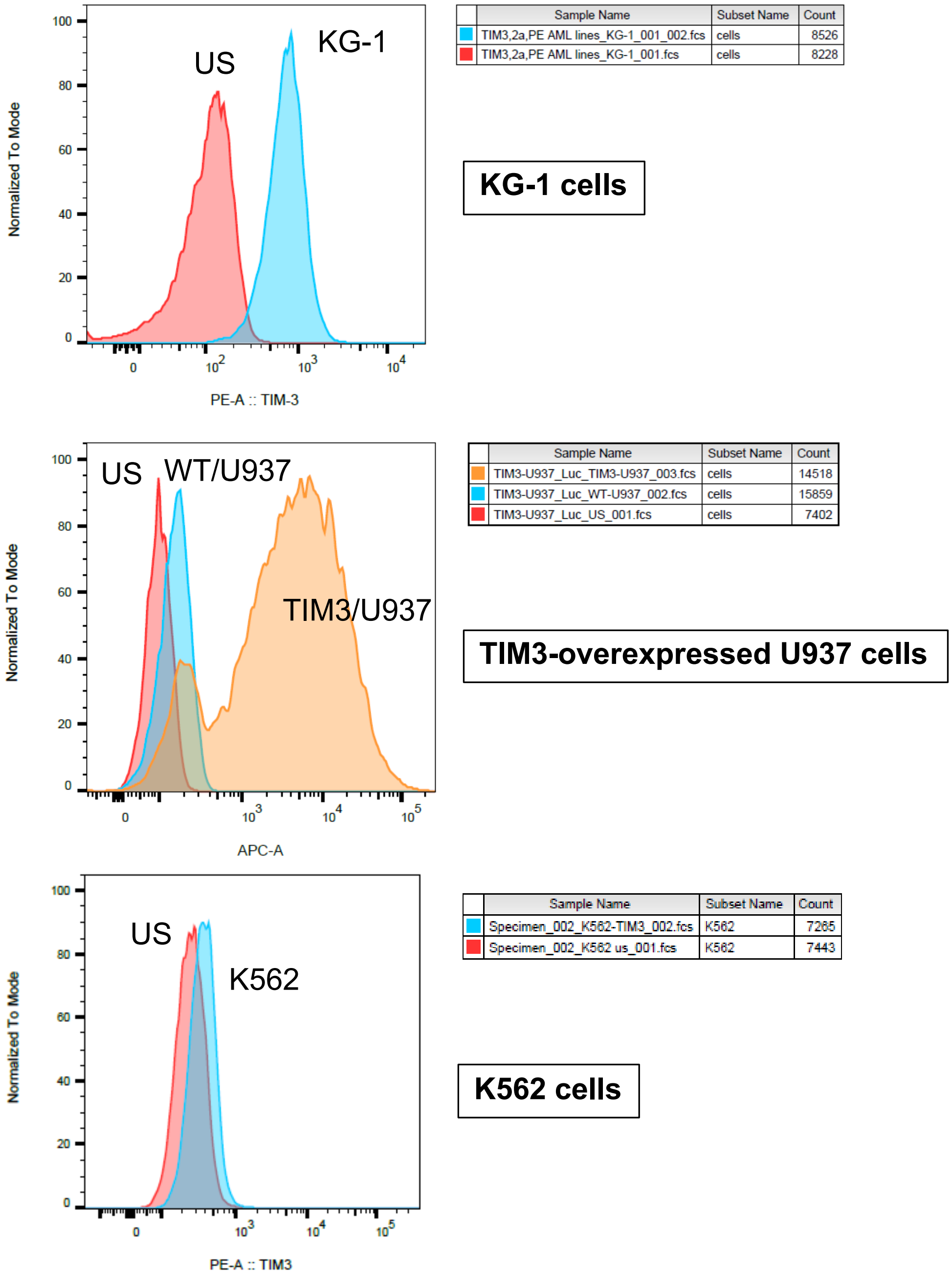

Suppl Fig S2 Analysis of TIM3 surface expression by flow cytometric analysis.

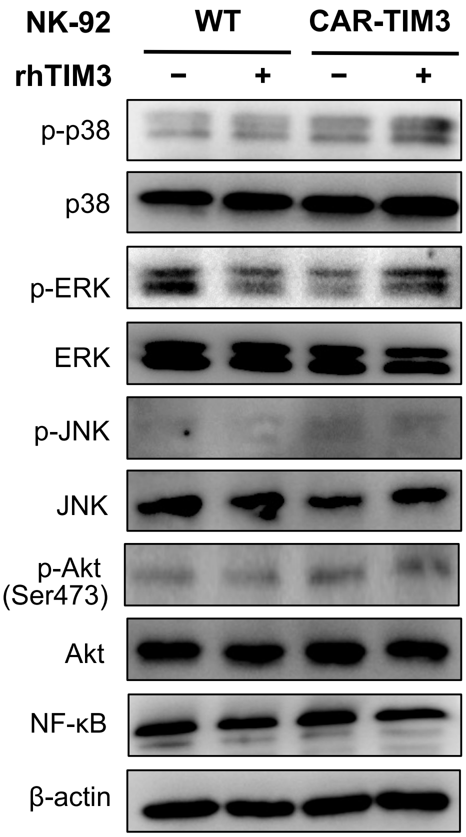

Suppl Fig S3 Western blot analysis of various intracellular signaling pathways in CAR-TIM3 NK-92 cells in response to recombinant human TIM3 (200 μg/mL) at 4 hours in comparison to WT NK-92 cells.
